# Walking out of the light verb jungle: Exploring the translation strategies of light verb constructions in Chinese–English consecutive interpreting

**DOI:** 10.3389/fpsyg.2023.1113973

**Published:** 2023-03-15

**Authors:** Di Wang, Guiying Jiang, Yufan Zheng

**Affiliations:** ^1^College of Foreign Languages and Cultures, Xiamen University, Xiamen, China; ^2^School of the English Language and Culture, Xiamen University Tan Kah Kee College, Xiamen, China

**Keywords:** light verbs, light verb constructions, translation strategies, Chinese-English consecutive interpreting, variability of strategy selection

## Abstract

Cross-linguistic features of light verb constructions (LVCs) profile a major facet of the typological difference between Chinese and English. By adopting a theory-driven, context-based interpreting task, this study explores the effectiveness and variability of translation strategies in dealing with 12 target LVCs extracted from a Chinese–English Consecutive Interpreting test to capture effective translation strategies fit for Chinese English-as-foreign-language (EFL) learners (*N* = 66). Appropriate rates and entropy values denoting variability of strategy selection are calculated by using 12 LVC segments and nine strategies, respectively. A correlation test is also carried out for vocabulary knowledge and the appropriate rates of LVCs to assess the efficacy of learners’ vocabulary knowledge in interpreting performance. Results show the general preferences for strategy selection among Chinese EFL learners as well as typical structural patterns in LVC translation. The degree of lightness of the light verbs exerts a reverse effect on the appropriate rates and consistency of strategy selection, and the positive correlation between vocabulary knowledge and LVCs’ appropriate rates suggests the need to incorporate the constructional teaching into the EFL learning curriculum. Thus felicitous conditions of applying the strategies have been proposed.

## 1. Introduction

Light Verb Constructions (LVCs; Grimshaw and Mester, 1988; Rosen, 1989; Butt, 1995; etc.) are basically constituted by a semantically bleached verb (e.g., make, have, get, give) and the action nominal complement (e.g., *answer, advice, help*), most typicssally denoting a motion event or state as in (1a). Its meaning is equivalent to the counterpart synthetic verb derived from the nominal complement as in (1b). In addition, LVCs are analogous to the ditransitive clause in syntactic form as in (1c).

(1) a. Mike gave a kiss to his mother.
  b. Mike kissed his mother.
  c. Mike gave a book to his brother.

This construction exhibits a cross-linguistic feature, such as Mandarin Chinese (Huang et al., 2014), Japanese (Grimshaw and Mester, 1988), Indo-European languages (e.g., Butt, 1995; Golshaie, 2016; Sundquist, 2018), etc. The common features mainly include: (1) verbo-nominal combination; (2) semantically semi-compositional; (3) ‘light’ verbs; and (4) ‘heavy’ nominal complements.

Yet cross-linguistic studies suggest that the licensing conditions of LVCs vary from language to language. Nagy et al. (2019) focus on the automatic detection of LVCs in four languages, namely, English, German, Spanish and Hungarian; they generalize both common and specific linguistic features from a typological perspective distinguished by five categories, namely, statistical, lexical, morphological, syntactic, and orthographic. Furthermore, the difference can be detected across regional varieties of English. Ronan and Schneider (2015) carried out an automated parser-based study to detect LVCs by inter-variety comparison in the Great Britain component and Ireland component in International Corpora of English. In their study, light verbs with higher frequency are more often used in British English, whereas light verbs with lower frequency tend to be more actively used in Irish English (Ronan and Schneider, 2015). Similar cross-regional investigations of Asia English versions have been carried out by Hoffmann et al. (2011) and Mehl (2017), but show different results in onomasiological preferences. These previous studies illustrate that exploring the common and specific features of LVCs across languages or varieties is weighted toward LVC detection in machine translation and L2 acquisition.

Similar to English, Chinese possesses a robust distribution of LVCs (Huang, 2009), especially in registers, such as official public speeches, legislation and science and technology texts (Wang and Zhang, 2014). As two typologically different languages, English and Chinese have fundamental differences that are embodied in cross-linguistic features of LVC usage, such as the presence/absence of inflectional markers, flexibility of modifications, position of PPs, fixedness of the combinations, etc. These discrepancies increase the difficulties in acquisition for Chinese EFL learners, especially evident in training their interpreting skills, which require the intellectual capacity to instantly transform idioms, colloquialisms and collocations into the equivalent information in the target language. Available Chinese–English comparative studies in LVCs are mainly carried out by Chinese researchers in the field of comparative linguistics, such as semantic properties (Chou, 2019), formal features applied in NLP (Wang and Zhang, 2014; Bai and Xue, 2015); syntactic formation in relation to argument structures (Zhu, 2019), etc. These studies compare five properties of LVCs from different aspects in the two linguistic systems. However, despite their prevalence in both languages, LVCs have not received much attention as formulaic sequences in EFL learners’ translations. The fixity of LVCs is presented as a gradient ranging from rigid to free, depending on the syntactic variability and the degree of lexical opacity. Most of the LVCs are not fixed enough to qualify as idioms, but the combinations of the components and modification used are constrained. The semantic complexities of light verbs show “greater cross-linguistic variability than nominal one” (Foley, 2010, p. 84). Such variability across the two languages might be the main cause of difficulties faced by Chinese EFL learners, which attracts interest in the inappropriate usage of LVCs and motivates explorations of corresponding strategies. Light verbs and other high frequency verbs are thought to be a significant barrier for EFL learners because of their limited knowledge (Altenberg and Granger, 2001). However, such knowledge of LVCs is limited in terms of Chinese–English translation strategies in the context of consecutive interpreting performance. Opting for appropriate strategies of the interpretation during learning can save processing and production efforts when retrieving the translation equivalents (McDonald and Carpenter, 1981; Gile, 1995), thus effortlessly bringing about better performance. In addition, locating LVC segments in the production of interpreting text-based sources may truly reflect LVCs usage in a linguistic context. Considering the gaps in the literature and necessity of the research in L2 language acquisition, this study aims to investigate effective translation strategies fit for Chinese EFL learners based on a Chinese–English consecutive interpreting (CECI) test. The study addresses the following three questions:

- What are the common translation strategies adopted by the professional interpreters when treating LVC segments in consecutive interpreting?
- How are translation strategies distributed when Chinese EFL learners deal with LVCs in the consecutive interpreting test?
- How does L2 learners’ vocabulary knowledge of English LVCs affect the translation of the target LVC segments in the consecutive interpreting test?

The answers to these questions can help Chinese EFL learners better understand LVCs in both languages and improve their performances in interpreting.

## 2. Literature review: Comparative studies on LVCs between English and Chinese

In this study, the literature review primarily concentrates on comparative studies on LVCs between Chinese and English because translation strategies of LVCs between these two languages have not been addressed in previous studies. This review can serve as the foundation for the discussion of translation strategies by identifying the typical syntacto-semantic features of LVCs in the two target languages.

Studies on English light verbs can be traced back to nearly a century ago. Poutsma (1929) described complex predicate constructions as ‘group verbs’, including but not limited to light verb constructions, which since then has begun to be noticed for its syntacto-semantic idiosyncrasy. The term ‘light verb’ was first coined by Jespersen (1954, p. 117) to denote semantically low-content verbs, and ‘light verb construction’ has since become a commonly accepted term for a bipartite complex predicate (e.g., Grimshaw and Mester, 1988; Rosen, 1989; Butt, 1995, 2003, 2010). Major disputes rest on whether the verb must be pertinent to an isomorphic (zero-derived) form (e.g., *drink* in *to have a drink*), a derivative (e.g., *decide* (v.)—*decision* (n.) in *to make a decision*), or a verbal noun (e.g., *effort* in *to make an effort*; e.g., Wierzbicka, 1982; Quirk et al., 1985; Algeo, 1995; Allerton, 2002; Dixon, 2005), and whether the direct object in the construction must be analysed as verbs or as nouns (e.g., Hoffmann et al., 2011).

Studies on Chinese LVCs occurred much later and has reached no consensus on terminology and classification of the light verb. Some use ‘formal verb’ to highlight its purely formal functions, as the meaning of the light verb is impoverished with no semantic contribution to the clause (e.g., Lv, 1980; Fan, 1981). Others (e.g., Yuan and Xia, 1984; Zhu, 1985) proposed ‘delexical verb’ or ‘quasi-predicate verb’, holding that the nominal complement collocating with a light verb must be a two-syllable verb-derived noun or modifier-head combination. Recent Chinese LVC studies mainly center on its syntactic representation and semantic features within Chomskyian generative linguistic sphere (Wen and Cheng, 2007; Zhang, 2013).

A limited number of Chinese–English comparative studies by Chinese researchers concentrate on delineating formal features of LVCs between the two languages (Wang and Zhang, 2014; Bai and Xue, 2015; Zhu, 2019). Under the guidance of Hierarchical Network of Concepts Theory (Huang, 1998), Wang and Zhang (2014) targeted Chinese–English machine translation of LVCs, taking the typical Chinese light verb *jìnxíng* ‘make’ type as an example. Three linguistic factors have been found to modulate the choice of syntactical structure in Chinese–English translation as follows: the semantic category of the light verb’s nominal complement, the syntactical form of LVCs in the target language, and the function of LVCs in the clause where they are located. Three translating rules have been put forward, but their effectiveness and applicability need to further testing. Bai and Xue (2015) attempted to distinguish predicative verbs with vague meaning from the true light verbs by analyzing syntactic and semantic features and argument assignments of the latter. This approach is analogous to Kearns (2002), in which the true light verb (e.g., *give a groan*) and the vague action verb (e.g., *give a demonstration*) are differentiated by testing their passivisation, WH-movement, and pronominalisation, etc. These two studies imply that both languages are involved in the issue of delimitation of the light verb, and syntactic and semantic functions of the nominal complement are the key determining factors to distinguish these two kinds of verbs.

The issue of delimitation of the light verb is aligned with grouping their different types by means of shared semantic attributes. Bai and Xue (2015, p. 11) rank the degree of ‘lightness’ of five major types of Chinese predicative verbs as shown in (2) (from light to heavy), and thus must be treated differently when annotated.

(2) GIVE > CAUSE = DO > BE > BECOME.

In the same vein, Feng (2016, p. 141) attempted to sequence five types of Chinese light verbs in terms of the degree of grammaticalization as in (3).

(3) CAUSE > TAKE/GET > DO > BE > BE (become/be-with)

Though the two orderings partly overlap, they differ in the research materials, naming of the light verb groups, testing methods, and semantic classification of major types; consequently, the results are different. Similarly, Zhu (2019, p. 155–156) classifies light verbs into four groups, namely, DO, CAUSE, CONSIDER, PREP (a provisional term, indicating a type of covert light verbs that can be used with prepositional phrases), but without sequencing likewise. Furthermore, the naming method that mixes syntactic and semantic attributes obscures classification.

Notably, Feng’s (2016) finding mainly comes from his substantial diachronic analysis of old Chinese (OC) compared with modern Chinese (MC). Interestingly, compared with LVCs in MC, those in OC are more similar to those in English (Feng, 2016, p. 114). For example, the light verbs in both OC and English are silent (without phonetic realization), which trigger syntactic shifts and give rise to semantic changes *via* denominalisation or causativisation, as shown in (4–5) (Huang, 2009, p. 2–3):

(4) English and OC denominals: *yú* ‘fish or to fish’, *shí* ‘food or eat’, *fàn* ‘rice or have rice’, *yī* ‘clothes or to clothe’, *yǐn* ‘drink or to drink’, etc.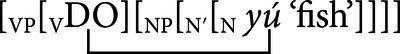.
(5) English and OC causatives: *bài ‘*lose or defeat’, *pò* ‘break’, *hǎo* ‘good or to like’, *wáng ‘*king, to regard as a king’, etc.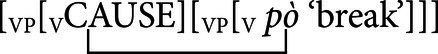.

However, in MC, the silent light verb becomes overt and fills the hypothesized position originally occupied with a silent category (Huang, 2009, p. 2). Thus, the examples in (4–5) can be instantiated by a light verb *dǎ* ‘hit’ in MC as in (6).

(6) *dǎ yú dǎ fàn dǎ bài dǎ pò*
hunt for fish buy meal hit defeat hit break
‘to do fishing to buy meals to cause to defeat to cause to break’
(Huang, 2009, p. 2)

As the two languages evolve, their typological differences emerge. For example, the analytic/synthetic account reveals that English has a large number of bound morphemes to denote the word property. Several action nominal complements in English LVCs are derived from verbs with bound morphemes, such as *contribute* (v.) converted into (*make a*) *contribution* (n.), or *investigate* (v.) into (*give an*) *investigation* (n.), while Chinese uses more free morphemes and has no inflectional markers to distinguish the parts of speech. As such, the nominal complements in Chinese LVCs are often in isomorphic (zero-derived) form. For example, *diàochá* (n.) ‘investigation’ in (2a) and *diàochá* (v.) ‘investigate’ in (7b) are identical in form.

(7) a. *zuòle yíge diàochá*
make-ASP one-CL investigation
‘made an investigation’
b. *diàochále yíge ànzi*
investigate-ASP one-CL case
‘investigated a case’

In addition, paratactic/hypotactic difference indicates that the relationship between the components or clauses is loose and flexible in the Chinese language but conforms to strict order with connectives in English. In Example (8), the theme argument introduced by the Chinese prepositional case marker *duì* ‘to’ can be placed either before (8a) or after (8b) the subject. However, the syntactic structure in (8b) is not acceptable in English. Such difference can be explained by topic−/subject-dominant account, that topic plays a fundamental role in Chinese clausal constructions. In addition to the canonical syntactic structure SVO like that in English, SOV and OSV are also prevalent in Chinese.

(8) a. *CSI duì zhège ànzi jìnxíngle diàochá*.
CSI to this-CL case proceed-ASP investigation.
‘CSI made an investigation of this case.’
b. *duì zhège ànzi, CSI jìnxíngle diàochá*.
to this-CL case CSI proceed-ASP investigation.
‘*Of this case, CSI made an investigation.’
(Kuo and Ting, 2007, p. 351)

Such flexibility is similarly exemplified by ‘separable LVCs’, i.e., a verbo-nominal combination is at times embedded with other attributive components, showing greater syntactical flexibility.

(9) *zuò-le yí-gè quánmiàn-de zǒngjié*
make-ASP one-CL comprehensive-MOD summary
‘give a comprehensive summary’

In (9), *quánmiàn* ‘comprehensive’ separates the light verb *zuò* ‘make’ and its nominal complement *zǒngjié* ‘summary’. This term can also be substituted by more modifiers, either simple adjectives or complex attributive clauses. However, such property varies in degree of flexibility and accessibility among different types of light verbs. Kuo and Ting (2007) divided the light verbs into two types, namely, MAKE group (e.g., *jìnxíng ‘*proceed’, *zuò* ‘do’) and GIVE group (e.g., *jiāyǐ* ‘give’, *yùyǐ ‘*give’, *gěiyǔ* ‘give’). It is claimed that only the insertion of modification by the MAKE group is allowed, and not with the GIVE group. In general, the common type of modification– such as articles, quantifiers, possessors, or adjectives–is placed before the nominal complement in Chinese LVCs. By contrast, Claridge (2000) dichotomizes English LVCs into LV + NP pairings and LV + NP + PP, such as *to run the risk of*. Unlike English, Chinese has no post modifier PP like that in English. The similar component in Chinese LVC sentences often functions as a theme argument projected by the deverbal noun, introduced with or without a preposition. For example,

(10) a. *gōng’ānjú duì zhège ànjiàn jìnxíngle diàochá*
The police office to_prep._ this-CL case proceed-ASP investigation
‘The police office proceeded an investigation to this case.’
b. *gōng’ānjú jìnxíngle zhège ànjiàn-de diàochá*
The police office proceed-ASP this-CL case-MOD investigation
‘The police office proceeded an investigation to this case.’

In (10a), the PP *duì zhège ànjiàn* ‘to this case’ comes before the complex predicate. By comparison, in (10b), the theme argument *zhège ànjiàn* ‘this case’, as a pre-nominal modifier, is placed directly before the nominal complement *diàochá* ‘investigation’.

While Chinese LVCs allow more diversified modifiers, the article usage, modification, and pluralization in English LVCs tend to be more fossilized (Brinton, 2008). Such phenomena can be traced back to the Middle English period, during which adjectival modifications were confined to a small range of adjectives (Matsumoto, 1999, p. 83). For several combinations, no modification has been identified, i.e., LVCs in this situation are lexicalized into fixed expressions, such as *take effect*, *lose sight of*, *give rise to* (Claridge, 2000, p. 157–158). However, Claridge (2000, p. 158) also indicated that, though rather rare, modification is found in well-established units such as *take full place*, or *find so much fault*, because the noun is salient and independent enough to be modified.

In addition, syntactic operation such as passivisation can render the light verb and its nominal complement separable and inverted, and is sometimes treated as a testing method to distinguish true light verb from vague action verb as mentioned earlier (Saito and Hoshi, 2000; Kearns, 2002; Kuo and Ting, 2007). Notably, the light verb and its nominal complement in a true light verb construction cannot be passivized. Such property is observed in both Chinese and English.

Another issue relates to the semantic difference between an LVC and its simplex predicate verb. It is generally agreed that LVCs are ‘semantically more lightweight than the same word would have been in a normal context’. (Allerton, 2002, p. 172) It is the same case in Chinese as is shown in example (11).

(11) a. *zuò-le yí-gè quánmiàn-de zǒngjié*
make-ASP one-CL comprehensive-MOD summary
‘give a comprehensive summary’
b. *zǒngjié-de hěn quánmiàn*
summarize-PAR very comprehensively-MOD
‘to summarize fully’

In (11a), *Quánmiàn-de* ‘comprehensive’ means that an overall summing-up has been completed while *quánmiàn* ‘comprehensively’ in (11b) denotes one typical property of the action ‘summarize’. For the record, not all nominal complements can be converted into the counterpart synthetic verb. For example, the change of *effort* into *to make an effort* cannot be used in the form *to effort. In this case, *make* is a light verb and *effort* denotes an abstract event. Chinese also has a group of specialized event nouns that cannot be converted into the counterpart synthetic verbs, such as *zhànzhēng ‘*warfare’, *yíshì* ‘ceremony’, *shoǔshù* ‘surgery’ (Lu, 2012).

In summary, from a broader perspective, the canonical order of LVCs basically shared by Chinese and English is ‘the light verb + nominal complement’, with or without modifiers such as articles, quantifiers, possessors, adjectives inserted in between. Besides, both languages are characterized by the categorization of true light verbs and vague action verbs, classification of event nouns, semantic differentiation between an LVC and its simplex predicate verb. The differences are reflected mainly in the presence or absence of inflectional markers, flexibility of modifications, position of PPs, fixedness of the combination, etc. These differences pose difficulties in the comprehension of LVCs and lead to various problems in conversion across languages. The present investigation attempts to be carried out with this line of research to delve into translation strategies of LVCs in the CECI test.

## 3. Research methodology

### 3.1. Research design

To capture the effective translation strategies fit for Chinese EFL learners, we follow a theory-driven, top-down procedure in this study. Two criteria are set, which are the guiding principles of extracting LVCs and the common translation strategies extracted from the professional interpreting work as the baseline for comparison. By comparing Chinese EFL learners’ performance with professional interpreting work, the predilection of translation strategy selection can be exhibited.

### 3.2. Participants

Sixty-six juniors (all are Chinese natives) majoring in English from a comprehensive university in China are selected for the study. The score of the National Test for English Major Grade 4 (henceforth TEM4) taken by the end of the second academic year is adopted to measure the participants’ general English proficiency. The passing rate of the participants is 57% (*N* = 66, Mean score = 61.92, SD = 7.90), slightly higher than the national average level (=52.69%). To guarantee normal distribution of the target data, we eliminate scores that are three standard deviations above or below the mean as outfielders. At the time of taking the interpreting test designed for the study, the participants had taken 2 years’ interpreting training, and are thus presumed to have generally acquired basic interpreting skills, including taking notes, memorizing strategies, analyzing, reconstructing languages. The study protocol is approved by the ethics review board of the university where the tests are carried out. Written informed consent is obtained from all participants. All of the procedures are performed in accordance with the Declaration of Helsinki and relevant policies in China.

### 3.3. Materials

The testing material is a public speech at a press conference and derived from available resource in the public domain^1^. The length of the speech is approximately 3 min, 20 s with 550 Chinese characters. The speed of speech is moderate and the articulation of the speaker is clear.

To provide a full picture of the common strategies adopted in the authentic context of interpreting, we manually collect the translating strategies adopted by the professional interpreters from a self-built small-size Chinese–English parallel corpus of speeches at press conferences held by China’s Ministry of Foreign Affairs from 1 July to 31 July, 2021 (85,495 Chinese characters, 64,456 English words, 2,658 sentence pairs). The materials are openly accessible in^2^.

### 3.4. Procedure

The interpreting test was arranged in an audio classroom. The test was arranged as one part of the final exam of the interpreting course at the time. The participants were first fully informed with instructions by the course teacher. Participants were allowed to take notes while listening to the soundtrack. The play was paused when the speech reached the natural end, and the participants then began to translate in the target language. The translation works were automatically recorded.

Given the importance of determining whether the related LVC expressions in target language are ready for use in interpreting, an after-test questionnaire about vocabulary knowledge is arranged right after the interpreting test to ensure consistency of the experiment. The items in the questionnaire are all related to the possible English versions of the 12 target LVC segments. The participants are expected to respond with their knowledge about those items. Based on the “vocabulary knowledge scale” designed by Paribakht and Wesche (1997), each item is rated in five scales: a. I have never met the expression before; b. I have seen it before, but I do not know its meaning; c. I have seen it before, and I think I may know its meaning; d. I know it. Its meaning is___ (paraphrase or translation); e. I know how to use it to make up a sentence, for example (if you choose this one, please fill in the blank in d, too.) The full design of the after-test questionnaire is presented in Appendix 1.

### 3.5. Extraction of target LVC segments and annotation principles

Given the limited consensus on the defining features—such as lightness of the light verb, properties of the nominal complement, and degree of modification—the current study follows three guiding principles in selecting and comparing relevant LVCs for the empirical analysis. The principles are generally acknowledged by previous LVC studies in both Chinese and English (Nagy et al., 2019; Zhu, 2019):

- The choice of target LVCs is limited to the overt light verb and nominal complement combination, considering the types of modification;
- The light verb is grammaticalized to the extent that attributes its semantically lighter meaning and major syntactical function to the construction as a whole;
- The nominal complement typically denotes an action or an event, assigning theta-roles of the arguments in the clause.

We firstly generate a frequency wordlist of verbs (*n* = 1,424) by using the online word parsing and processing tool *Weiciyun*^3^. Two coders use the three guiding principles to select the light verbs from the wordlist exhaustively and generated 928 concordance lines that were manually scrutinized as target LVC segments in Mandarin in the self-built Chinese–English parallel corpus translated by professional interpreters. Their English counterparts are marked for retrieving translation strategies. Finally, nine strategies are set as the benchmark for comparison with learners’ versions, as listed below:

Type A: Literal translation (or transliteration).
LV + (MOD) + N -- > LV + (MOD) + N.

(12) *dáchéngle zhòngyào gòngshí*
reach-ASP important common understandings
‘reached important common understandings’

Literal translation is preferred when an English LVC equivalent to the Chinese counterpart is available. However, most cases are far more complex due to cross-linguistic differences. Therefore, further translation strategies are necessary to meet the needs, which are illustrated as follows:

Type B: VP conversion.
LV + (MOD) + N -- > N-derived V (+MOD).

(13) *zuòle yígè héxīn guīnà*
do-ASP one-CL core summary
‘summarized them up into one thing’

Type C: Verb missing.
LV + (MOD+) N -- > (MOD+) N.

(14) *bǎochí wěndìngde shuāngbiǎn(zhōngměi) guānxī*
maintain steady-MOD bilateral (China-US) relationship
‘steady growth of China–US relationship’

Type D: Passive voice.
LV + (MOD+) N -- > (MOD+) N + be + LV-ed.

(15) *qǔdéle fēngshùo chéngguǒ*
get-ASP a great deal accomplishments
‘a great deal has been accomplished’

Type E: Inverted LVC.
N + (MOD+) LV -- > (MOD+) N + LV (+MOD).

(16) *máodùn jiūfēn yě jīngcháng tūchūde biǎoxiànchūlái*
Conflict disputes too often distinctly-MOD present out-COMP
‘from time to time problems and difficulties may have occurred’

Note that Type E is derived from a canonical LVC with the light verb and its nominal complement inverted to form an unaccusative clause, and the verb is often modified by an adverb of degree indicating the gradient property. As both Chinese and English have such syntactic structure, literal translation can be used.

Type F: Copula construction.
LV + N1(MOD) + N2 -- > N1(MOD) + be + N2.

(17) *bǎochí zhōngměi guānxi zǒngtǐ wěndìng*
keep China–US relationship general stable
‘China–US relationship is stable’

Type G: There be construction.
LV + N1(MOD) + N2 -- > There be + N2 + Prep. + N1(MOD).

(18) *jìnxíng yìmiáo hùrènde tǎolùn*
proceed vaccine mutual recognition-MOD discussion
‘there are discussions on mutual recognition of vaccines’

Type H: PP conversion.
LV + N -- > Prep. + N.

(19) *zuòle yígè héxīn guīnà*
do-ASP one-CL core summary
‘in conclusion’

Type I: DUI argument shifting.
DUI-NP1 + LV + NP2 -- > NP2-derived V + NP1.

(20) *duì xiāngguān jízhuāngxiāng jìnxíngle xiūfù he qiánghuà*
to_prep._ Relevant containers proceed-ASP repair and strengthen
‘(it) has repaired and strengthened the relevant containers’

The nine strategies summarized above are used as the baseline to identify learners’ deviation from the standard or appropriate translation strategies and to observe the different preferences in dealing with LVCs.

In line with the three guiding principles for selection, we also extracted 12 Chinese LVC segments categorized into three major types by their semantic attributes from the testing material, namely, DO, BE, BECOME (Feng, 2005, 2016) from the testing material. Table 1 show the segment classifications.

**Table 1:** Target Chinese LVC segments (L1–L12).

NO	CH	EN	TYPE
L1	zuòle yígè héxīn guīnà	Sum them up	DO
	Make-ASP one-CL core summary		
L2	qǔdéle fēngshuò chéngguǒ	A great deal has been accomplished	BECOME
	Get-ASP rich achievement		
L3	yǒuzhe guǎngfànde gòngtóng lìyì	There is a broad common interest	BE
	Have-ASP broad-MOD common interest		
L4	bǎochí wěndìngde shuāngbiān guānxi	Steady growth of China-US relationship	BE
	Keep steady-MOD bilateral relationship		
L5	bǎochí zhōngměi guānxi zǒngtǐ wěndìng	Maintaining the overall stability of China-US relationship	BE
	Keep China-US relationship overall stability		
L6	máodùn jiūfēn yě jīngcháng tūchūde biǎoxiàn chūlái	Problems and difficulties may have appeared	BECOME
	Problem dispute too often prominently-MOD present out-COMP		
L7	dáchéngle zhòngyào gòngshí	Reached important common understandings	BECOME
	Reach-ASP important common sense		
L8	jìnxíng chuōshāng	Consultations between the two sides on economic and trade issues are still under way	BE
	Make consultation		
L9	shíxiàn hùlì gòngyíng	Deliver win-win and mutual benefits to the two countries	BECOME
	Achieve mutual benefit win-win		
L10	tuīdòng zhōngměi guānxide fāzhǎn	Continue to grow China-US relationship, including their economic and trade ties	BECOME
	Promote China-US relationship-MOD development		
L11	jìnxíng huàjiě guǎnkòng	Defuse their differences and manage them properly	BE
	Proceed solution control		
L12	tuīdòng fǔhé shìjièchάoliúde zhōngměiguānxi wěndìng fāzhǎn	Pursue steady and sound growth of China-US relationship	BECOME
	Push fit world trend-MOD China-US relationship steady growth		

The English version provided here is translated on site by a Chinese professional interpreter.

The testees’ interpreting works are recorded and transcribed after the test (about 22,000 words in total). The basic information listed in the transcribed texts includes the student number, name, class, and test score. In addition, 12 target LVC segments of each testee’s version are manually tagged by the types of strategy as stated earlier in this section, and separately marked with tick or cross to indicate translating appropriateness by the two raters. The general plan is to track the proportion of frequency and the acceptability rate of each strategy type adopted by the 12 target LVC segments in response to the first two research questions.

### 3.6. Reliability

The evaluation criterion of this test follows the assessment for Chinese undergraduate students based on interpreting process (Chen, 2017). To guarantee the reliability of the result, two professional interpreting teachers were invited as raters to evaluate the appropriateness of target LVCs. A reliability test is carried out and a high degree of consensus (*r* = 0.967) shows to ensure the consistency of the rating system. In cases of disagreement, a third independent rater can be invited to determine discrepancies to settle the disagreements.

### 3.7. Analyzing methods

Entropy computation is used to measure variability by the 12 LVC segments and the nine strategies, in addition to the regular descriptive statistics to summarize the general features of the current data set, The entropy H of a variable quantifies the degree of randomness or variability (Cover and Thomas, 2005). The formula is presented as below:


$$H\left(x\right)=\overset{n}{\underset{x=1}{\sum }}p\left(x\right)\log\;p\left(x\right)$$

For the 12 LVC segments, *x* denotes each of the nine strategies, and *p(x)* is estimated with the proportion of strategies that participants have adopted for translating a given LVC segment. The entropy score per LVC segment is a measure of how diverse strategies are used to translate a given LVC segment. The entropy value close to zero indicates either the translating strategies used for a given LVC segment is relatively consistent. In contrast, high entropy means more options have been taken in treating a given LVC segment.

For the nine strategies, *x* denotes each of the 12 target LVC segments, and *p*(*x*) is estimated with the proportion of target LVC segments that a given strategy is applied to. The entropy score per strategy is a measure of consistency of strategy applications. The entropy value close to zero indicates the strategy is consistently applied to relatively fewer limited LVC segments. In contrast, high entropy implies that the strategy is employed by a wider range of LVC segments.

Entropy enjoys increasing use in the language sciences (e.g., Montemurro and Zanette, 2011; Gries, 2012) for its preponderance in variability computation. It can be used for computing both categorical and continuous variables. In addition, it is comparable across individuals and categories due to a specific quantification of the variability value.

In answer to the third research question, a correlation test is carried out between English vocabulary knowledge and the appropriate rates of LVCs in the interpreting test.

## 4. Results

In response to the three research questions, three sets of data are reported: (1) distribution of translation strategies (including the proportion of frequency and entropy values) used by the participants for the 12 LVC segments; (2) the appropriate rates of the 12 LVC segments and the nine translation strategies, as well as their interrelation with the semantic attributes of light verbs; and (3) the correlation between vocabulary knowledge of LVCs and the interpreting score of the 12 target LVC segments.

**Figure 1:**
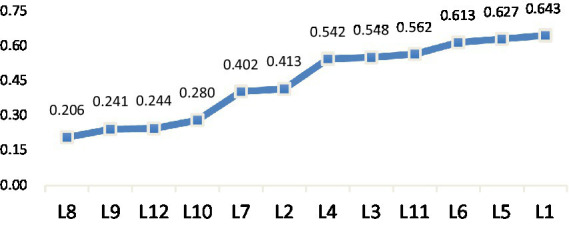
Entropy value by LVC segments.

**Figure 2:**
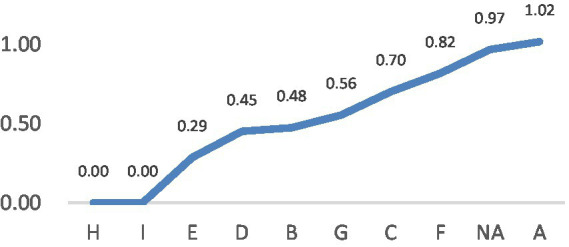
Entropy value by strategies.

In general, literal translation type (A) takes up the highest proportion in selection (54.92%), followed by Types C (10.61%), B (6.44%) and G (6.31%; see Table 2). In terms of entropy value by LVC segments (Figure 1), the scores of L8, 9, 10, 12 are relatively low, which implies that when they are translated into English, the translation strategies used by the participants are relatively consistent and mostly centralized in Type A. These LVC segments mainly pertain to BE and BECOME grouped by semantic attributes of the light verb. By contrast, L1 (DO group) shows the highest entropy (*H* = 0.643) indicating high variability in strategy selection distributed in almost all strategies except Types E and F. For entropy value by strategies (Figure 2), the scores of Types H, I and E are relatively low, implying that those strategies are applied to a limited number of LVC segments. By contrast, Type A shows the highest entropy (*H* = 1.02) indicating its wide application in various LVC segments.

**Table 2:** Raw data of translation strategies for the 12 LVC segments.

_LVC_^strategy^	A	B	C	D	E	F	G	H	I	NA^a^	Total
L1	21 (13)	14 (12)	6 (4)	1 (1)	0 (0)	0 (0)	3 (3)	0 (0)	0 (0)	21 (0)	66 (33)
L2	43 (29)	0 (0)	0 (0)	0 (0)	0 (0)	5 (1)	3 (2)	0 (0)	0 (0)	15 (0)	66 (32)
L3	38 (35)	0 (0)	10 (4)	0 (0)	1 (1)	2 (0)	9 (8)	0 (0)	0 (0)	6 (0)	66 (48)
L4	35 (30)	1 (0)	16 (14)	1 (1)	0 (0)	4 (2)	0 (0)	0 (0)	0 (0)	9 (0)	66 (47)
L5	34 (28)	2 (2)	8 (6)	0 (0)	0 (0)	11 (11)	3 (1)	1 (1)	0 (0)	7 (2)	66 (51)
L6	11 (9)	0 (0)	1 (1)	0 (0)	14 (11)	8 (4)	30 (20)	0 (0)	0 (0)	2 (0)	66 (45)
L7	25 (19)	1 (1)	37 (34)	0 (0)	0 (0)	1 (0)	0 (0)	0 (0)	0 (0)	2 (0)	66 (54)
L8	56 (41)	0 (0)	0 (0)	0 (0)	0 (0)	0 (0)	1 (0)	0 (0)	0 (0)	9 (0)	66 (41)
L9	57 (45)	0 (0)	5 (1)	0 (0)	1 (0)	0 (0)	1 (0)	0 (0)	0 (0)	2 (0)	66 (46)
L10	55 (47)	1 (1)	0 (0)	0 (0)	1 (0)	5 (2)	0 (0)	0 (0)	0 (0)	4 (0)	66 (50)
L11	4 (0)	31 (24)	1 (1)	2 (0)	0 (0)	0 (0)	0 (0)	0 (0)	24 (9)	4 (0)	66 (34)
L12	56 (40)	1 (1)	0 (0)	0 (0)	0 (0)	3 (2)	0 (0)	0 (0)	0 (0)	6 (0)	66 (43)
Total	435 (336)	51 (41)	84 (65)	4 (2)	17 (12)	39 (22)	50 (34)	1 (1)	24 (9)	87 (2)	792 (524)
Percent (%)	54.92	6.44	10.61	0.51	2.15	4.92	6.31	0.13	3.03	10.98	100

The correct number is shown in the bracket.

a NA refers to omissions in interpreting.

**Figure 3:**
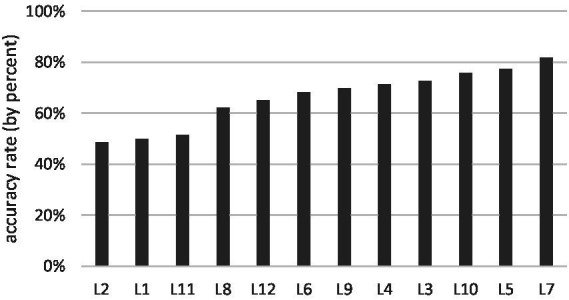
The appropriate rates of the 12 LVC segments (by percent).

**Figure 4:**
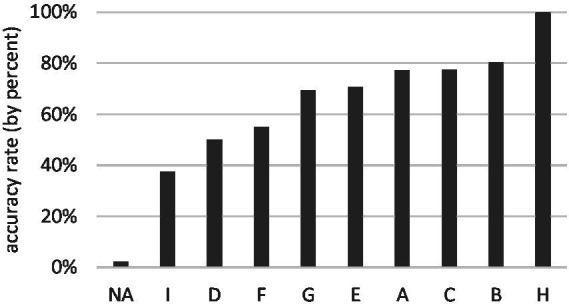
The appropriate rates of the nine translation strategies (by percent).

The appropriate rates of the 12 LVC segments (Figure 3) and of the nine translation strategies (Figure 4) are unevenly distributed. A relatively higher rate is achieved in L3, 4, 5, 7, 10 and in Types A, B, C, respectively, (given that only one case is translated as Type H, the result can be ignored).

**Figure 5:**
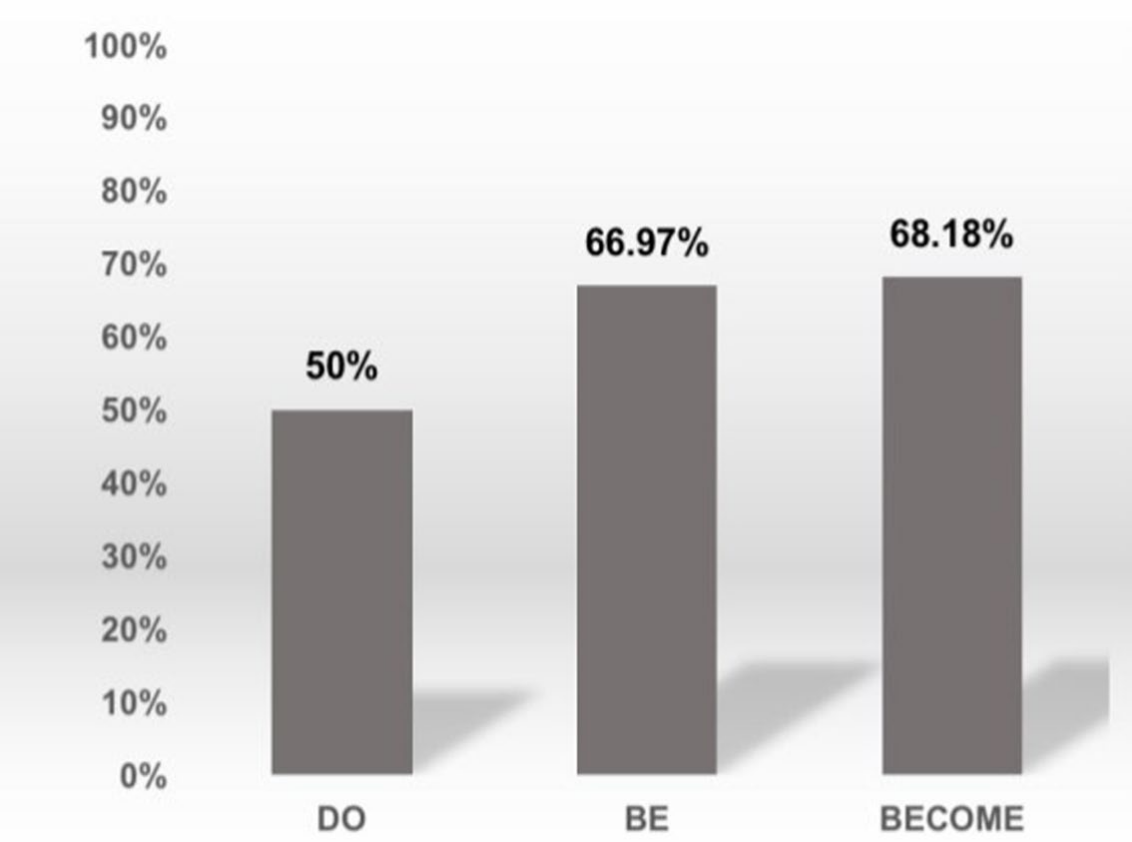
The appropriate rates grouped by semantic attributes.

To obtain a closer look at the appropriate rate by semantic attribute groups, we compute the grand means of appropriate rate using the three semantic attribute groups, as shown in Figure 5. The BECOME group has the highest rate, and the DO group has the lowest.

We also compute the grand means of entropy value by semantic attribute groups in a similar manner, and the results are given in the reverse order than those of the appropriate rates (Mean_DO_ = 0.643; Mean_BE_ = 0.497; Mean_BECOME_ = 0.366). These results indicate that as the light verb becomes more abstract, the translation appropriate rate decreases and the variability increases (or consistency decreases) in strategy selection. In summary, the degree of lightness of the light verbs exerts a reverse effect on the appropriate rate and consistency of strategy selection.

Furthermore, a correlation test is carried out between the English vocabulary knowledge related to the 12 target LVCs and the appropriate rates of the 12 target LVC segments in the interpreting test. Given that both of the testing variables are normally distributed, and the assumption of linearity is not markedly violated, Pearson correlations are computed to examine the two variables. The result shows a significant correlation (*r* = 0.272, *p* < 0.05). According to Cohen (1988), the vocabulary knowledge and the appropriate rates of the 12 target LVC segments have a positive correlation, which is considered as a close medium effect size (*r* = 0.3). This finding means that students with a good command of target topic-related lexical knowledge are likely to have high LVC scores in the interpreting test.

## 5. Discussion

In this section, under the background of typological differences and syntactic and semantic properties of the construction, we mainly discuss the achieved results from three motivating factors proposed by the research questions: a. preferences of strategy selection; b. structural patterns in LVC translation; and c. relations of lexical knowledge and appropriate use of LVCs. Thereupon, the felicitous conditions of the nine translation strategies are summarized along with related pedagogical implications.

### 5.1. Preferences of strategy selection

Different processing inclinations are identified and discerned by observing the participants’ preferences for different translation strategies in treating LVCs during the C-E interpreting test.

Based on our observation from the data, the most frequently-used strategy is literal translation. The reason is obvious: literal translation saves the processing effort so as to allocate more attention where necessary. However, the misuse of light verbs frequently occurs, as examples in (21):

(21) a. * ‘to have an conclusion’
‘to draw a conclusion’
b. * ‘to move stable and healthy relationship’
‘to maintain stable and healthy relationship’

Without knowledge of an equivalent LVC, the testee would preferably choose Types B or C, which are retaining the nominal complement only or converting the noun into a verb, respectively. However, the potential problem of choosing Type C is that, when the light verb is removed, no predicate is left in the clause. If the testee cannot find a syntactic predicate for the nominal complement at the time, an unacceptable expression may be produced. Here is an ill version in (22):

(22) *tuīdòng fǔhé shìjiè chάoliúde zhōngměi guānxi wěndìng fāzhǎn*
push fit world trend-MOD China-US relationship steady growth
*‘…the world relationship and our development to the health and steady…’

In the translated version of L12, only a nominal complement *fāzhǎn ‘*development’ is retained. The testee cannot reorganize the sense relations of the source LVC segment with limited processing capacity, and fails to properly translate the light verb *tuīdòng* ‘push’ and the complex attribute clause that modifies the noun phrase.

The choice of Type B indicates that the participants assume that LVCs and their counterpart synthetic verbs are interchangeable. If no matchable LVC is available for conversion, a nominal-derived verb might be used. However, different from the simplex verbal predicate, the light verb in LVCs is proposed to serve an aspectual function (Wierzbicka, 1982), and thus the two forms are actually not identical. However, a corpus study indicates that it is the ease and variety with the usage rather than the semantic minuscule difference that motivates the use or disuse (Bonial and Pollard, 2020). The data of the current study validate the statement, given that the choice of Types B or C shows no regular tendency.

Besides Types B and C, Type G (*There be construction*) is another frequently-used strategy in translation of the target LVC segments, especially in L6, but its appropriate rate seems low (=63.18%), as in (23):

(23) *máodùn jiūfēn yě jīngcháng tūchūde biǎoxiànchūlái*
problem dispute too often prominently-MOD present out-COMP
‘There are conflicts and disputes that arise frequently’

This unaccusative clause is inverted from a canonical verbo-nominal word order. The statistics shows that a preferable option to translate such derived LVC is *There-be construction* for lack of a volitional subject. However, the misuse of the construction mainly lies in confounding the existential predicate *There be* with the light verb. If a testee fails to arrange the two into an appropriate hierarchical structure, then errors will follow, such as:

(24) *‘there have a lot of conflicts’
*‘there are appear some disputes’
*‘there are still some frictions emerge’

### 5.2. Structural patterns in LVC translation

The statistics indicate that the appropriate rates of the 12 LVC segments (Figure 3) are relatively high in L3, 4, 5, 7,10, and low in L1, 2, 6, 8, 9, 11, 12. Further observation of participants’ performances reveals that the main reason for mistranslation is the differences of modification patterns in the two languages. Though internal grammatical modification is a common feature in both Chinese and English LVCs, the intricate difference may cause inappropriate translation.

One major difference is that Chinese LVCs allow more diversified modifiers, while English LVCs tend to be more fossilized. The tighter connection of such LVCs becomes more unified such as a simplex verb, increasing the probability of taking a post adverbial component. In this case, strategy B is often adopted in C–E conversion. That is to say, the Chinese separable LVCs with adjectival modification in between may be converted into a synthetic verb counterpart with a post adverbial modification in English as in (11), if the target language has no equivalent LVC available in the target language. The LVC segment L1 selected from the testing material is a similar case, as illustrated in (24).

(25)a. *zuò-le yí-gè héxīn guīnà*
make-ASP one-CL core-MOD summary.
‘sum them up’.
b. *do a core summary.

In 25(a), the verb phrase ‘sum up’ is used to translate the LVC expression in the professional version. However, a participant who fails to find the appropriate equivalence adopts a literal translation and overused the delexical verb ‘do’ to form an unacceptable LVC expression. Moreover, cautious consideration is needed in that prenominal modifications in Chinese LVCs are not always adjectival but rather may be nominal or an expression with dual semantic properties, such as *héxīn* ‘core’ in Example (25). The nominal modification functions as a genitive case and its thematic role is assigned by the verbal noun rather than the light verb. For example:

(26) *bǎochí zhōngměi guānxi zǒngtǐ wěndìng*
keep China-US relationship overall stability
‘maintaining the overall stability of China–US relationship’

In (26), the verbal noun *wěndìng* ‘stability’ (NP2) retains its semantic relationship with the nominal modifier *zhōngměiguānxi ‘*China–US relationship’ (NP1), forming an internal semantic relation--assigner (NP2) - assignee/patient (NP1). When converting into English, the common treatment is to leave NP1 behind NP2 led by a preposition, i.e., LV + NP2 + Prep. + NP1. Given that post modification is rare in Chinese NP, if an L2 learner is not aware of this cross-linguistic distinction, then an erroneous translation is likely to occur in limited working time.

Another major difference in this regard is that Chinese LVCs are at sometimes separated by prenominal adjectival components. However, in English, the presence/absence of the prenominal adjectival modification may be affected by frequency and syntactic fixity of the collocation. The nominal modifier in a Chinese separable LVC is frequently represented by ‘DUI-insertion’ (Zhu, 2019). ‘DUI’ represents all the prepositional case markers, such as *duì* ‘to’, *duìyú* ‘as for’, *bǎ* ‘about’, or *gēn* ‘along with’. This approach is equivalent to the function of a preposition inserted in an English LVC, introducing the patient of the verbal noun. ‘DUI’ is also often inserted between the subject and the LVC, but sometimes it may move forward to the head as the topic of the sentence as in (27).

(27) *duìyú máodùn hé fēnqí jìnxíng huàjiě guǎnkòng*
as for conflict and disagreements proceed solution control

‘As for the differences and disagreements, we have confidence to defuse their differences and manage them properly.’

In this sentence, the theme argument *máodùn hé fēnqí* ‘differences and disagreements’ of LVC introduced by a preposition *duìyú* ‘as for’ is topicalized and projected by the LVC segment *jìnxíng huàjiě guǎnkòng ‘*proceed solution and control’. In fact, the topicalized theme argument semantically functions as the direct object of verbal noun phrase *huàjiě guǎnkòng* ‘solution and control’. However, different from Chinese which is a topic-prominent language, English is subject-prominent. Hence, the common practice in dealing with the above Chinese LVC is to translate into a canonical SVO English structure with the verb derived from the nominal complement and the object attained from a prepositional argument. Example (20) of Type I illustrates this approach. Our data show that the appropriate rate of this strategy is only 37.50%. In addition, the participants do not favour Type I (*n* = 24; percentage≈3.03%), which implies that most testees are not used to applying the strategy into C–E interpreting.

Another observation is that most testees prefer attributive to adverbial modifications in their English versions. The main reason is that literal translation is most frequently used than other strategies. In Chinese, an attributive modification is normally placed before its head noun, and the English version is likely to follow such word order. Similar preference is also discussed by Fleischhauer and Neisani (2020) in Persian separable LVCs: although their study focuses on a different language, their findings present significance in understanding LVCs in general. Like Persian, many Chinese adverbials are either overtly or non-overtly derived from adjectives, as given in Example (11). No inflectional marker is available to use to distinguish adjectives and adverbials, which share identical lexical forms that are not clear-cut in most cases. One typical test for distinction is to use the post-modification particle *de* (的) for adjectives and *de* (地) for adverbials. The internal attributive modifications in Chinese LVCs are mainly regarded as adjectives. However, internal modifiers in LVCs do not always share similar semantic functions with those in counterpart synthetic verbs, as L7 shows in (28).

(28) *dáchéngle zhòngyào gòngshí*
reach-ASP important common understanding
‘reached important common understandings’

Some argue that the internal modifiers in Chinese LVCs basically modify the whole construction rather than the nominal components. However, in (27), ‘important’ modifies the nominal component ‘common understandings’, for the adjective specifies the importance of understanding, i.e., the mutual goal shared by the political leaders is crucial for the future friendly negotiation. This part cannot be paraphrased as ‘understand importantly’. Therefore, learners need to know the felicitous conditions of the modification in the target language to achieve proper translation.

### 5.3. Relations of lexical knowledge and appropriate use of LVCs

The correlation test performed between the target LVCs knowledge and the appropriate rates of the 12 target LVC segments shows a significant positive result. Thus EFL learners with a good command of target topic-related lexical knowledge are very likely to have high LVC score in the interpreting test. Furthermore, the grand means of entropy value by semantic attribute groups shows that the degree of lightness of the light verbs exerts a reverse effect on the appropriate rate and consistency of strategy selection. Specifically, as the light verb becomes more delexicalized, the appropriate rate in translation decreases, and the variability increases (or consistency decreases) in strategy selection.

This study groups 12 Chinese LVC segments into three types based on their semantic attributes, i.e., DO, BE, BECOME, illustrated in Table 1. According to Feng (2016), the degree of grammaticalization can be ordered from high to low as in (28) below.

(29)DO>BE>BECOME

The appropriate rates grouped by semantic attributes (Figure 5) are consistent with the order of the degree of grammaticalization: the former decreases as the latter increases, and vice versa. This result implies that the lightness of the light verb affects the equivalence of interpreting. In addition, the entropy value computed in the same fashion further indicates that the lightness of the light verbs might affect strategy selection. Being semantically light yet functionally complex, such light verb can have multiple treatments in translation. Given that most light verbs are polysemous, and LVCs are typical for their complex predicates with complex meanings, unskillful learners may be easily confused in discriminating ‘light’ usage from the ‘heavy’ sense. Naturally, intermediate EFL learners may have difficulties in such indirect and obscure matching process. As argued by Butt (2010), the light verb in LVCs contributes a generic meaning rather than an actual motion concept, namely, a full verb. Therefore, the degree of lightness of the light verbs exerts a reverse effect on the appropriate rates and consistency of strategy selection.

### 5.4. Felicitous conditions of the strategies and implications on formulaic language learning

**Figure 6:**
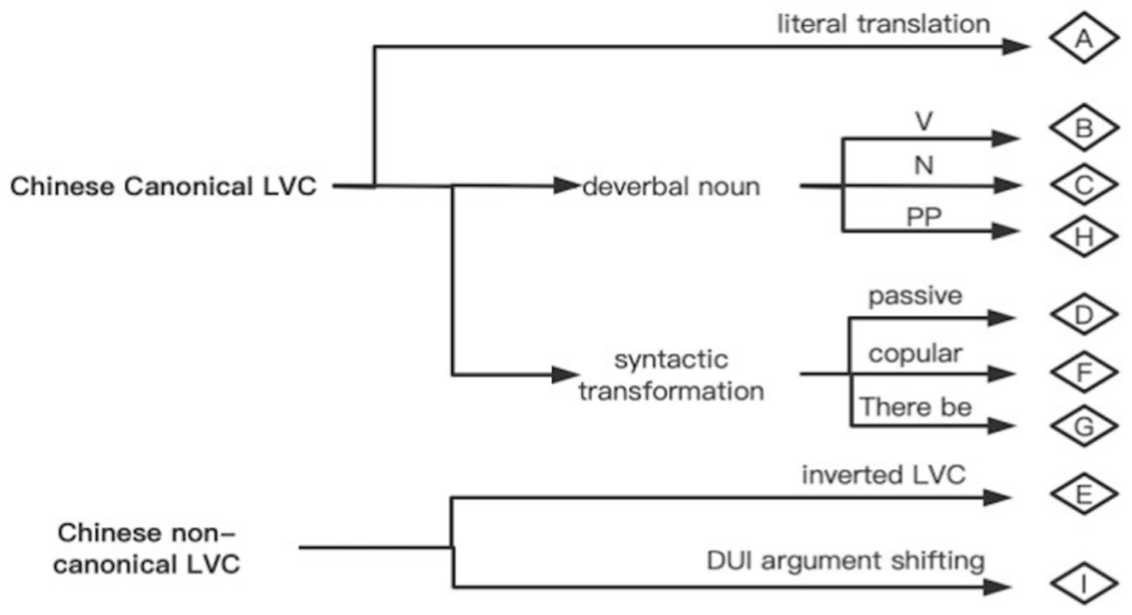
Felicitous conditions of LVC translation strategies.

Considering the three aspects discussed above, applicable conditions of the nine LVC strategies are briefly shown in Figure 6.

If an LVC expression is available in both Chinese and English, then Type A is the best option. If no direct equivalence is available in the target language, then the major semantic bearer—the verbal noun—may play a key role in the conversion (Types B, C, H), or syntactic transformation may be considered (Types D, F, G). Both Chinese and English have inverted LVC, and thus literal translation (Type E) can be used. As for the special case of Chinese non-canonical LVC with DUI-insertion, a canonical SVO English structure (Type I) can be used with the verb derived from the nominal complement and the object attained from the prepositional argument.

In addition to the common translating issues illustrated above, other minor problems occur in this test, such as morphological misuse (especially between the nominal verbs and deverbal nouns), syntactic variation of LVCs (such as passivity in L6), and PP shift (as illustrated in Types H and I). Moreover, the strategy selections illustrated above are not mutually exclusive, but work together in translation.

Apart from major linguistic factors, one’s formulaic knowledge may also affect the selection of translation strategies. The proper use of formulaic sequences in the target language may bridge the gap between native speakers and L2 learners (e.g., Wray, 2002; Wood, 2010), and a few illuminating attempts to quantify pattern variability of fomulaicity in text registers (e.g., Roemer, 2010; Forsyth and Grabowski, 2015). Langacker (2008) considered ‘formulaic representation’ as the basic tool to coginitively understand language. The concept of ‘slate’ in language is represented in various linguistic levels such as pronunciation, lexical, syntax. These modular slate structures are believed to be the smallest unit of English language communication. Formulaicity forms a gestalt as a holistic representation. As mentioned in Section 5.2, English LVCs tend to be more fossilized in contrast to those of Chinese. The fixed combination calls for L2 learners’ awareness of formulaicity in the target language. Formulaic sequence is neither a word nor a syntactical structure but a lexical–syntactical continuum to construct a text. It has low decomposability and high cohesion, which renders it considerable advantages in bilingual transformation. However, most frequency-driven approaches have not yet been widely applied in translation and interpreting studies. Possibly, different formulaic constructions are treated with no difference. The correlation test between the after-test questionnaire on vocabulary knowledge and the LVCs appropriate rates in the current study supports the claim that formulaic use improves fluency and accuracy of interpreting by helping to alleviate the limited cognitive load and promote his processing capacity to save time (Tang and Li, 2013, 2016).

## 6. Conclusion

This study explores the appropriateness and variability of translation strategies in dealing with the 12 target LVC segments by L2 learners to capture effective translation strategies fit for Chinese learners of English in this regard. The results show that the frequency of use and the appropriate rates of nine types of translation strategies are distributed unevenly; as such, preference of strategy selection conforms to the economy principle, i.e., saving the processing effort to allocate more attention to wherever necessary. Moreover, the consistency between the appropriate rates and of strategy selection grouped by semantic attributes and the order of the degree of grammaticalization implies that the lightness of the light verb affects the appropriateness and strategy selection of translation, i.e., the degree of lightness of the light verbs exerts a reverse effect on the appropriate rates and consistency of strategy selection. Meanwhile, the positive correlation between the after-test questionnaire and interpreting score shows that a good command of target topic-related lexical knowledge helps to improve the interpreting performance. The teacher needs to determine whether the L2 learners have or have not appropriately acquired the target expressions to determine whether it is the strategy itself or unfamiliarity with the target expression that leads to failure in bilingual translation. The felicitous conditions of the nine translation strategies are thus delineated. The findings support that cultivating the awareness of formulaicity and acquiring translation strategies help L2 learners develop a set of matching system across languages, and improve fluency and accuracy of C-E interpreting.

As an attempt to explore the interpreting strategies of LVC, the current study only offers data observed from Chinese to English translation, and not the other way around. A full picture can be obtained if bidirectional interpreting tasks can be designed. We hope that future studies will further probe into the issue by providing more comprehensive experimental support.

## Data availability statement

The raw data supporting the conclusions of this article will be made available by the authors, without undue reservation.

## Ethics statement

The studies involving human participants were reviewed and approved by Xiamen University Tan Kah Kee College. The patients/participants provided their written informed consent to participate in this study.

## Author contributions

DW: the conception and design of the work, the acquisition, analysis, and interpretation of data for the work, drafting the original version. GJ: the conception and design of the work, revising the draft critically for important intellectual content. YZ: data collection, revising the draft critically. All authors contributed to the article and approved the submitted version.

## Funding

This work was supported by the Humanities and Social Science General Program sponsored by the Ministry of Education of the People’s Republic of China (grant number 18YJA740047).

## Conflict of interest

The authors declare that the research was conducted in the absence of any commercial or financial relationships that could be construed as a potential conflict of interest.

## Publisher’s note

All claims expressed in this article are solely those of the authors and do not necessarily represent those of their affiliated organizations, or those of the publisher, the editors and the reviewers. Any product that may be evaluated in this article, or claim that may be made by its manufacturer, is not guaranteed or endorsed by the publisher.

## Footnotes

^1^http://www.gov.cn/gongbao/content/2019/content_5377102.htm

^2^https://www.fmprc.gov.cn/

^3^www.weiciyun.com

## Abbreviations

ASP: aspectual particle
C-E: Chinese-English
CECI: Chinese-English consecutive interpreting
CL: classifier
COMP: complement
L: LVC segment
LV: Light Verb
LV-ed: the past participle form of the light verb
LVC: Light Verb Construction
MOD: modifier
MC: modern Chinese
N: noun
NA: not available
NP: noun phrase
OC: old Chinese
PAR: particle
Prep.: preposition
PP: prepositional phrase
SD: standard deviation
TEM4: Test for English Major Grade 4
V: Verb
VK: Vocabulary knowledge

## References

[ref1] Algeo J. (1995). “Having a look at the expanded predicate,” in The verb in contemporary English: theory and description. eds. Aarts B. Meyer C. F. (Cambridge: Cambridge University), 203–217.

[ref2] Allerton D. J. (2002). Stretched verb constructions in English. London: Routledge.

[ref3] Altenberg B. Granger S. (2001). The grammartical and lexical patterning of MAKE in native and non-native student writing. Appl. Linguist. 22, 173–195. doi: 10.1093/applin/22.2.173

[ref4] Bai X. Xue N. (2015). A study on argument structure of ‘V + event NP-Obj’ construction in Chinese from a multi-lingual perspective. J. Foreign Lang. 38, 2–13.

[ref5] Bonial C. Pollard K. A. (2020). Choosing an event description: what a PropBank study reveals about the contrast between light verb constructions and counterpart synthetic verbs. J. Linguist. 56, 577–600. doi: 10.1017/S0022226720000109

[ref6] Brinton L. J. (2008). “Where grammar and lexis meet,” in Theoretical and empirical issues in grammaticalization. eds. Seoane E. López-Couso M. J. (Amsterdam: John Benjamins), 33–53. doi: 10.1075/tsl.77.04bri

[ref7] Butt M. (1995). The structure of complex predicates in Urdu. San Antonio, TX: CLSI Stanford.

[ref8] Butt M. (2003). “The light verb jungle,” in Papers from the GSAS/Dundley house workshop on light verbs (Harvard working papers in linguistics 9). eds. Aygen G. Bowern C. Quinn C. (Cambridge, MA: Harvard University Press), 1–50.

[ref9] Butt M. (2010). “The light verb jungle: still hacking away” in Complex predicates: cross-linguistic perspectives on event structure. eds. Amberber M. Baker B. Harvey M. (Cambridge, UK: Cambridge University Press), 48–78.

[ref10] Chen Y. (2017). Assessment standard construction in the course of consecutive interpreting for undergraduates oriented from interpreting process. TEFLE 144, 74–81.

[ref11] Chou W. (2019). A comparative study of the English and Chinese Delexical construction from the perspective of cognitive linguistics. Shanghai, China: Shanghai Foreign Language Education Press.

[ref12] Claridge C. (2000). Multi-word verbs in early modern English: a corpus-based study. Amsterdam: Rodopi.

[ref13] Cohen J. (1988). Statistical power analysis for the behavioral sciences. (2nd ed.). Hillsdale, NJ: Erlbaum.

[ref14] Cover T. M. Thomas J. A. (2005). Elements of information theory. Hoboken, NJ: Wiley.

[ref15] Dixon R. M. W. (2005). “She gave him a look, they both had a laugh and then took a stroll: give a verb, have a verb and take a verb constructions,” in A semantic approach to English grammar. ed. Dixon R. M. W. (Oxford: Oxford University Press), 459–483.

[ref16] Fan X. (1981). A general account of verb in Chinese. Shanghai: Shanghai Education Press.

[ref17] Feng S. (2005). Light verb movement in modern and classical Chinese. Linguist. Sci. 14, 3–16.

[ref18] Feng S. (2016). A preliminary theory of diachronic syntax in Chinese. Shanghai: Shanghai Educational Publishing House.

[ref100] Fleischhauer J. Neisani M. (2020). Adverbial and attributive modification of Persian separable light verb constructions. J. Linguistics. 56, 45–85. doi: 10.1017/S0022226718000646

[ref200] Foley W. A. (2010). “Events and serial verb constructions” in Complex Predicates: Cross-linguistic Perspectives on Event Structure. eds. M. Amberber, B. J. Baker, and M. Harvey (Cambridge University Press), 79–109.

[ref19] Forsyth R. S. Grabowski L. (2015). Is there a formula for formulaic language? Poznań Stud. Contemp. Linguist. 51, 511–549. doi: 10.1515/psicl-2015-0019

[ref20] Gile Daniel (1995). Basic concepts and models for interpreter and translator training. Philadelphia, PA: John Benjamins.

[ref21] Golshaie R. (2016). A corpus study on identification and semantic classification of light verb constructions in Persian: the case of light verb xordan ‘to eat/collide’. Lang. Sci. 57, 21–33. doi: 10.1016/j.langsci.2016.05.002

[ref22] Gries S. (2012). Frequencies, probabilities, and association measures in usage−/exemplar-based linguistics: some necessary clarifications. Stud. Lang. 36, 477–510. doi: 10.1075/sl.36.3.02gri

[ref23] Grimshaw J. Mester A. (1988). Light verbs and θ-marking. Linguist. Inquiry 19, 205–232.

[ref24] Hoffmann S. Hundt M. Mukherjee J. (2011). Indian English an emerging epicentre? A pilot study on light verbs in web-derived corpora of south Asian Englishes. Anglia J. English Philol. 129, 258–280. doi: 10.1515/angl.2011.083

[ref25] Huang Z. (1998). HNC (hierarchical network of concepts) theory. Beijing: Tsinghua University Press.

[ref26] Huang C.-T. J. (2009). Lexical decomposition, silent categories, and the localizer phrase. Essays Linguist. 39, 86–122.

[ref28] Huang C. Lin J. Jiang M. Xu H. (2014). “Corpus-based study and identification of mandarin Chinese light verb variations,” in Proceedings of the first workshop on applying NLP tools to similar languages, varieties and dialects. eds. Zampieri M. Tan L. Ljubešić N. Tiedemann J. (Dublin, Ireland: VarDial Workshop), 1–10.

[ref29] Jespersen O. (1954). A modern English grammar on historical principles, part VI: Morphology. London: Bradford and Dickens.

[ref30] Kearns K. (2002). Light verbs in English. Master’s Thesis. MIT.

[ref31] Kuo P. Ting J. (2007). “Light verb, heavy verb and verbal noun in mandarin Chinese,” in: *Proceedings of the 9th Seoul international conference on generative grammar (SICOGG 9), Seoul*, 349–357.

[ref32] Langacker R. W. (2008). Cognitive grammar: a basic introduction. New York: Oxford University Press.

[ref34] Lu B. (2012). The semantic characteristics of ‘event nouns’ in Chinese and English. Contemp. Linguis. 14, 1–11.

[ref35] Lv S. (1980). Eight hundred words in modern Chinese. Beijing: Commercial Press.

[ref36] McDonald J. L. Carpenter P. A. (1981). Simultaneous translation: idiom Interpreation and parsing heuristics. J. Verbal Learn. Verbal Behav. 20, 231–247. doi: 10.1016/S0022-5371(81)90397-2

[ref300] Matsumoto M. (1999). “Composite predicates in Middle English” in Collocational and idiomatic aspects of composite predicates in the history of English. eds. L. J. Brinton, and M. Akimoto (Amsterdam: John Benjamins), 59–95.

[ref37] Mehl S. (2017). Light verb semantics in the international corpus of English: onomasiological variation, identity evidence and degrees of lightness. English Lang. Linguist. 23, 55–80. doi: 10.1017/S1360674317000302

[ref38] Montemurro M. A. Zanette D. H. (2011). Universal entropy of word ordering across linguistic families. PLoS One 6:e19875. doi: 10.1371/journal.pone.0019875, PMID: 21603637 PMC3094390

[ref39] Nagy T. I. Rácz A. Vincze V. (2019). Detecting light verb constructions across languages. Nat. Lang. Eng. 26, 1–30. doi: 10.1017/S1351324919000330

[ref41] Paribakht T. S. Wesche M. B. (1997). Reading comprehension and second language development in a comprehension based ESL programme. TESL Canada J. 11, 09–27. doi: 10.18806/tesl.v11i1.623

[ref42] Poutsma H. (1929). A grammar of late modern English (part I: The sentence). Groningen: P. Noordhoff.

[ref43] Quirk R. Greenbaum S. Leech G. Svartvik J. (1985). A comprehensive grammar of the English language. London: Longman.

[ref44] Roemer U. (2010). Establishing the phraseological profile of a text type. The construction of meaning in academic book reviews. English text. Construction 3, 95–119. doi: 10.1075/etc.3.1.06rom

[ref45] Ronan P. Schneider G. (2015). Determining light verb constructions in contemporary British and Irish English. Int. J. Corpus Linguist. 20, 326–354. doi: 10.1075/ijcl.20.3.03ron

[ref46] Rosen S. T. (1989). Argument structure and complex predicates. PhD dissertation. Waltham, MA: Brandeis University.

[ref47] Saito M. Hoshi H. (2000). “The Japanese light verb construction and the minimalist program,” in Step by step: Essays on minimalist syntax in honor of Howard Lasnik. eds. Martin R. Michaels D. Uriagereka J. (Cambridge, MA: MIT Press), 261–295.

[ref49] Sundquist J. D. (2018). A diachronic analysis of light verb constructions in old Swedish. J. Ger. Linguist. 30, 260–306. doi: 10.1017/S1470542717000137

[ref50] Tang F. Li D. (2013). Explicitation in Chinese-English consecutive interpreting: A comparative study of professional and student interpreters. Foreign Lang. Teach. Res. 45, 442–452.

[ref51] Tang F. Li D. (2016). Explicitation patterns in English-Chinese consecutive interpreting: differences between professional and trainee interpreters. Perspectives 24, 235–255. doi: 10.1080/0907676X.2015.1040033

[ref53] Wang H. Zhang K. (2014). Light verb construction in Chinese-English translating study based on corpus--take ‘*jinxing*’ as an example. J. PLA Univ. Foreign Lang. 37, 62–68+144.

[ref54] Wen B. Cheng J. (2007). On syntactic nature of the light verb v. Modern Foreign Lang. 30, 17–20.

[ref55] Wierzbicka A. (1982). Why can you have a drink but you can’t have an eat? Language 58, 753–799. doi: 10.2307/413956

[ref56] Wood D. (2010). Formulaic language and second language speech fluency. Background, evidence and classroom applications. London: Continuum.

[ref57] Wray A. (2002). Formulaic language and the lexicon. Cambridge: Cambridge University Press.

[ref58] Yuan J. Xia Y. (1984). A general account of delexical verbs. Second. Lang. Res. 7, 31–40.

[ref59] Zhang Z. (2013). Syntax and semantic study of light verb. Foreign Lang. Educ. 34, 17–20.

[ref60] Zhu D. (1985). Delexical verbs and nominal verbs in modern written Chinese. J. Peking Univ. 5, 3–8.

[ref61] Zhu L. (2019). A contrastive analysis of light verbs in English and Chinese. Beijing: Foreign Language Teaching and Research Press.

