# Supplementary material for: Walking out of the light verb jungle: Exploring the translation strategies of light verb constructions in Chinese–English consecutive interpreting
Source: Front Psychol. 2023 Mar 15;14:1113973. doi: 10.3389/fpsyg.2023.1113973 (PMC10050874; doi:10.3389/fpsyg.2023.1113973)
Supplement: Supplementary file 2 [file Data_Sheet_2.docx]

# Appendix: Vocabulary Knowledge Questionnaire (Chinese Version)

学生姓名：　　　　　　　学号：　　 　　 作答时长：

短语理解程度问卷调查

亲爱的同学们好！

感谢你的宝贵时间配合完成“短语理解程度”问卷调查。本问卷仅供“英语短语熟悉度对汉英交替传译产出质量的影响”相关研究所使用，不是考试，不计入该科目考试成绩，无对错之分。我们很想知道你对相关题目的真实反应，希望能得到你的支持，以确保数据的可靠性。我们将对你所提供的宝贵信息严格保密。感谢配合！

请根据你对下列短语（或单词）的熟悉程度选择符合自身情况的选项并将相应题号写在括号内，每题含下列五个选项：

1. 我从没见过这个短语（或单词）;
2. 这个我见过，但不知道它是什么意思；
3. 这个我见过，好像知道它是什么意思；
4. 我认识它，它的意思是：　　　　　　　　　　　　　　　　　（翻译或释义均可）
5. 我知道如何使用它造句（在保证短语基本意思不变的前提下，可适当改变词性），比如：

　(如果你选择这一项，请同时填写选项D相应信息。)

（　）１. reach mutual understanding

释义：

造句：

（ ）2. consensus

释义：

造句：

（　）3.sum up to one thing

释义：

造句：

（　）4. make a generalization

释义：

造句：

（ ）5. steady growth of China-US relationship

释义：

造句：

（ ）6. maintain stable bilateral relations

释义：

造句：

（　）7.achievement

释义：

造句：

（ ）8.yield fruitful results

释义：

造句：

（ ）9. a great deal has been accomplished

释义：

造句：

（ ）10. extensive common interest

释义：

造句：

（ ）11. share mutual benefits

释义：

造句：

（ ）12. dispute

释义：

造句：

（ ）13. problems and difficulties may have appeared

释义：

造句：

（ ）14. negotiation

释义：

造句：

（ ）15. consult with

释义：

造句：

（ ）16. be in consultation

释义：

造句：

（ ）17. win-win strategy

释义：

造句：

（ ）18. realize mutual benefits

释义：

造句：

（ ）19. deliver win-win and mutual benefits to the two countries

释义：

造句：

（ ）20. economic and trade ties

释义：

造句：

（ ）21. promote the growth of

释义：

造句：

（ ）22. resolve

释义：

造句：

（ ）23. defuse differences and manage properly

释义：

造句：

（ ）24. prominent

释义：

造句：

# Vocabulary Knowledge Questionnaire (English Version)

Name：　　　　　　Student No.：　　 　　 Testing Length：

Dear Participants,

Thank you for taking a few minutes to complete the questionnaire below related to our research project on “the effect of familiarity with English phrases on the translation quality of Chinese-English consecutive interpreting”. This questionnaire is not a formal exam and will not be included in the exam score. They do not have standard answers. We would like to know your true response to the relevant questions and hope to get your support to ensure the reliability of the data. We will keep the valuable information you provide strictly confidential. Thank you for your cooperation!

According to your familiarity with the following phrases (or words), please choose the options that are suitable for your situation and fill in the brackets. Each item is rated on a scale of 1-5 as follows:

1. I’ve never met the expression before;
2. I’ve seen it before, but I don’t know its meaning;
3. I’ve seen it before, and I think I may know its meaning;
4. I know it. Its meaning is___ (paraphrase or translation);
5. I know how to use it to make up a sentence, for example (if you choose this one, please fill in the blank in 4, too.)

（　）1. reach mutual understanding

Meaning：

Sentence：

（ ）2. consensus

Meaning：

Sentence：

（　）3.sum up to one thing

Meaning：

Sentence：

（　）4. make a generalization

Meaning：

Sentence：

（ ）5. steady growth of China-US relationship

Meaning：

Sentence：

（ ）6. maintain stable bilateral relations

Meaning：

Sentence：

（　）7.achievement

Meaning：

Sentence：

（ ）8.yield fruitful results

Meaning：

Sentence：

（ ）9. a great deal has been accomplished

Meaning：

Sentence：

（ ）10. extensive common interest

Meaning：

Sentence：

（ ）11. share mutual benefits

Meaning：

Sentence：

（ ）12. dispute

Meaning：

Sentence：

（ ）13. problems and difficulties may have appeared

Meaning：

Sentence：

（ ）14. negotiation

Meaning：

Sentence：

（ ）15. consult with

Meaning：

Sentence：

（ ）16. be in consultation

Meaning：

Sentence：

（ ）17. win-win strategy

Meaning：

Sentence：

（ ）18. realize mutual benefits

Meaning：

Sentence：

（ ）19. deliver win-win and mutual benefits to the two countries

Meaning：

Sentence：

（ ）20. economic and trade ties

Meaning：

Sentence：

（ ）21. promote the growth of

Meaning：

Sentence：

（ ）22. resolve

Meaning：

Sentence：

（ ）23. defuse differences and manage properly

Meaning：

Sentence：

（ ）24. prominent

Meaning：

Sentence：
